# Accuracy and Consistency of Confidence Limits for Monosyllable Identification Scores Derived Using Simulation, the Harrell–Davis Estimator, and Nonlinear Quantile Regression

**DOI:** 10.3390/diagnostics14131397

**Published:** 2024-06-30

**Authors:** Vijaya Kumar Narne, Dhanya Mohan, Sruthi Das Avileri, Saransh Jain, Sunil Kumar Ravi, Krishna Yerraguntla, Abdulaziz Almudhi, Brian C. J. Moore

**Affiliations:** 1 Department of Medical Rehabilitation Sciences, College of Applied Medical Sciences, King Khalid University, Abha 61481, Saudi Arabia; sravi@kku.edu.sa (S.K.R.); ksuryanarayana@kku.edu.sa (K.Y.);; 2 Speech Language Pathology Unit, College of Applied Medical Sciences, King Khalid University, Abha 61481, Saudi Arabia; 3 Department of Speech Pathology and Audiology, Amrutha Institute of Medical Sciences, Kochi 682041, Kerala, India; dhanyam@aims.amrita.edu (D.M.);; 4 Department of Audiology/Prevention of Communication Disorders, All India Institute of Speech and Hearing, Manasagangothri, Mysore 570006, Karanataka, India; saranshjain@aiishmysore.in; 5 Cambridge Hearing Group, Department of Psychology, University of Cambridge, Cambridge CB2 1TN, UK; bcjm@cam.ac.uk

**Keywords:** confidence limits, quantile regression, pure-tone average, word identification scores

## Abstract

Background: Audiological diagnosis and rehabilitation often involve the assessment of whether the maximum speech identification score (PB_max_) is poorer than expected from the pure-tone average (PTA) threshold. This requires the estimation of the lower boundary of the PB_max_ values expected for a given PTA (one-tailed 95% confidence limit, CL). This study compares the accuracy and consistency of three methods for estimating the 95% CL. Method: The 95% CL values were estimated using a simulation method, the Harrell–Davis (HD) estimator, and non-linear quantile regression (nQR); the latter two are both distribution-free methods. The first two methods require the formation of sub-groups with different PTAs. Accuracy and consistency in the estimation of the 95% CL were assessed by applying each method to many random samples of 50% of the available data and using the fitted parameters to predict the data for the remaining 50%. Study sample: A total of 642 participants aged 17 to 84 years with sensorineural hearing loss were recruited from audiology clinics. Pure-tone audiograms were obtained and PB_max_ scores were measured using monosyllables at 40 dB above the speech recognition threshold or at the most comfortable level. Results: For the simulation method, 6.7 to 8.2% of the PB_max_ values fell below the 95% CL for both ears, exceeding the target value of 5%. For the HD and nQR methods, the PB_max_ values fell below the estimated 95% CL for approximately 5% of the ears, indicating good accuracy. Consistency, estimated from the standard deviation of the deviations from the target value of 5%, was similar for all the methods. Conclusions: The nQR method is recommended because it has good accuracy and consistency, and it does not require the formation of arbitrary PTA sub-groups.

## 1. Introduction

Speech identification scores (SISs) for phonetically balanced (PB) word or syllable lists are often included in an audiological test battery. If time permits, SIS can be measured at different sensation levels. The highest score obtained is denoted PB_max_ and it represents the best score for words in quiet that the patient can achieve when unaided. Due to time restrictions, SISs are commonly estimated in clinical practice at a single high level, chosen to be well above the detection threshold. The SIS at that level is also often denoted PB_max_ [1,2,3], although the true PB_max_ may be higher than this measured value. In addition to indicating a person’s receptive communication abilities, PB_max_ is useful in the differential diagnosis of auditory disorders and the fitting of hearing aids [4].

The pure-tone average (PTA) hearing threshold level (HTL), usually averaged across the frequencies 0.5, 1, 2, and 4 kHz, is the most commonly used index of peripheral hearing loss [5,6], and it has been used as a predictor of word scores, including PB_max_ values, in many studies [7,8]. Knowing if the PB_max_ obtained at a suprathreshold level is poorer than would be expected from the PTA is important when using PB_max_ for differential diagnoses [9,10]. For example, a poorer-than-expected PB_max_ might indicate a retro-cochlear disorder or auditory neuropathy. To determine whether the PB_max_ is lower than expected from the PTA, the lower boundary of the PB_max_ value expected for a given PTA must be estimated.

Yellin et al. [11] first proposed using the lower boundary for PB_max_ to determine whether the PB_max_ for an individual with sensorineural hearing loss was poorer than would be expected from the PTA. They referred to this as being “disproportionately poor”. They calculated the PTA as the average HTL across 1, 2, and 4 kHz. From the scatterplot relating PB_max_ to PTA, a lower boundary (a straight line relating PB_max_ to PTA) was constructed such that approximately 98% of the observed values fell above the boundary. A PB_max_ score was considered “disproportionately poor” if it fell below this empirically derived boundary. However, the relationship between PTA and PB_max_ was non-linear and many of the PB_max_ values were at the ceiling (100%). Due to this, it is inappropriate to determine the lower boundary of PB_max_ associated with a specific PTA by fitting a linear function to the data [7,8].

Dubno, Lee, Klein, Matthews, and Lam [7] adopted a different method for estimating the lower boundary of PB_max_ as a function of PTA. They considered 407 hearing-impaired ears from 212 participants, and divided them into 11 sub-groups based on PTA. The number of participants within each sub-group ranged from 18 to 50, which meant that the 95% confidence limit (CL) for each sub-group could not be determined accurately. To deal with this problem, a computer simulation method was used. The measured standard deviations (SDs) of the PB_max_ scores within each sub-group were always larger than the SDs estimated from the binomial distribution (see Equation (1) in Dubno, Lee, Klein, Matthews, and Lam [7]), on average by a factor of 1.62. Hence, for each PTA group, 2500 samples of normally distributed PB_max_ scores were generated using the mean determined from the experimental data and 1.62 times the SD estimated from the binomial distribution. Within each PTA sub-group, a one-tailed 95% CL was estimated from the simulation data. The values obtained in this way are denoted 95% CL_S_. A function was then fitted to the simulated 95% CL_S_ values to predict the 95% CL_S_ as a continuous function of PTA. Hereafter, this is referred to as the simulation method. The simulation method was adopted by Margolis et al. [12] for estimating 95% CL values for three sets of recorded speech materials, Q/MASS Tillman, VA, and Auditec [13].

In addition to the simulation method, several robust distribution-free methods have been proposed for estimating the 95% CL for non-normal distributions [14,15]. Such methods may be more accurate than the simulation method since the distribution of PB_max_ values varies with PTA and is rarely normal [7,8]. Among the various distribution-free methods, the Harrell–Davis (HD) estimator [16] performs better for small datasets and extreme quantiles [14,15,17]. Like the simulation method, the Harrell–Davis method requires the formation of sub-groups with different PTA values.

Non-linear quantile regression (nQR) [18,19] is another method that does not require assumptions about normality, and it has the additional advantage that it does not require the formation of sub-groups with different PTA values, which eliminates the sampling errors that occur with sub-grouping. Narne, Möller, Wolff, Houmøller, Loquet, Hammershøi, and Schmidt [8] used the nQR method to determine one-tailed 95% CL, denoted 95% CL_QR_, for a word list in Danish, using data from a large group of young and old participants with sensorineural hearing loss. They found that approximately 5% of the PB_max_ values fell below the estimated 95% CL_QR_ values, indicating that the method did converge on the target 95% CL.

The estimation of an extreme quantile like 95% is affected by the sample distribution, sample size, and the estimation method [17,20,21]. Previous studies have assessed the performance of a given method for estimating the 95% CL by measuring the percentage of PB_max_ values that fell below the estimated 95% CL; ideally, this should be close to 5%. However, another important aspect of the performance of a method for determining the 95% CL is its consistency. This can be estimated by applying the method to data obtained from different samples of patients and assessing how well the estimated 95% CL values agree across the samples.

The present study compared three methods for estimating the 95% CL as a function of PTA, namely simulation, HD, and nQR. The performance of each method was assessed using two measures: (1) how accurately the method estimated the 95% CL, i.e., how close to 5% was the number of PB_max_ values falling below the estimated 95% CL; (2) how consistent the method was in estimating 95% CL values when data from different samples of participants were used. To perform these analyses, the available dataset was randomly split into two, such that each dataset had 50% of the data. One set, called the fitting set, was used to estimate 95% CL as a function of PTA. The fitted function was then applied to the remaining 50% of the data, called the test set, and the proportion of cases falling below the fitted 95% CL was determined. This whole procedure was repeated 25 times, each with a different random draw of 50% of the data for the fitting set. The deviation (DEV) of the percentage of cases falling below the fitted 95% CL from the target value of 5% was determined. The mean of DEV across the 25 draws gives a measure of the accuracy and the SD gives a measure of the consistency.

The simulation and HD methods depend on the division of participants into PTA sub-groups. A final objective was to assess how this grouping affected the estimated 95% CL values. To do this, 95% CL was estimated using the simulation and HD methods for various PTA sub-groups.

## 2. Materials and Methods

### 2.1. Participants

Patients referred for hearing aid treatment with various degrees of hearing loss were recruited from the JSS Institute of Speech and Hearing, Mysore. All the participants underwent pure-tone audiometry and an otolaryngologic examination. It was determined that no participants required any surgical or medical treatment for their hearing loss apart from hearing aids. Initially, 720 participants were recruited. Seventy-eight participants had an air–bone gap ≥15 dB for at least two frequencies in the frequency range 0.5 to 2 kHz, indicating a conductive component of the hearing loss, and these were excluded, leaving 642 participants, 386 men and 256 women. Their ages ranged from 17 to 84 years with a mean of 52, median of 68, and SD of 20 years. Of these, 185 were older than 60 years and 447 were 60 years old or younger.

### 2.2. Procedure

#### 2.2.1. Basic Audiological Evaluation

All the audiological evaluations were carried out in double-walled sound-treated rooms. A calibrated two-channel diagnostic audiometer (Piano, Inventis, Padova, Italy) with TDH-39 headphones (Telephonics, Farmingdale, NY, USA) was used to determine the pure-tone air-conduction HTLs and a B-71 bone vibrator (Radioear, Middelfart, Denmark) was used for bone-conduction HTLs. HTLs were estimated using the modified Hughson–Westlake procedure [22]. The PTA was calculated for each ear separately by taking the average of the air-conduction HTLs at 0.5, 1, 2, and 4 kHz.

#### 2.2.2. Speech Recognition Threshold (SRT)

The speech recognition threshold (SRT) in quiet was measured in the same session as pure-tone audiometry using paired words developed by the All-India Institute of Speech and Hearing in the Kannada language. The stimuli were presented using monitored (via a sound-level meter) live voice via the Piano audiometer and Sennheiser HDA-200 headphones. The SRT was estimated separately for each ear using the method proposed by the American Speech–Language–Hearing Association [23]. A modified Hughson–Westlake method was used. After two or three correct answers out of three, the level was reduced by 10 dB. After 0 or 1 correct answer out of 3, the level was increased by 5 dB. The final SRT was taken as the lowest level giving 2/3 correct.

#### 2.2.3. Maximum Speech Identification Score (PB_max_)

PB_max_ was obtained for each ear using two phonemically balanced lists developed by Mayadevi and Vyasamurthy [24], each list containing 25 non-meaningful monosyllabic consonant–vowel (CV) stimuli. The second list used the same stimuli as the first list, but in a different randomized order. One list was used for each ear. The stimuli were presented through TDH-39 headphones using monitored live voice. Although recorded materials would be preferable for measuring PB_max_, many audiologists use monitored live voice presentations [2,3,25,26,27]. Testing began with the better-hearing ear (based on the PTA) for the participants with asymmetrical hearing loss; if the PTA was the same for the two ears, testing began with the right ear. The list was presented in quiet at 40 dB above the SRT measured in quiet or at the most comfortable level in cases of severe hearing loss, where loudness discomfort prevented the use of levels 40 dB above the SRT. Masking in the non-test ear was used when necessary according to standard masking rules [28].

#### 2.2.4. Statistical Analyses

All the statistical analyses were performed using the R software version 3.6 [29] and programmed with RStudio, V 2022.12 [30]. Quantile regression was performed using the “quantreg” package, V 5.95 [31]. Confidence limits using the HD method were estimated in the R software using the “WRS” package, V 0.40 [32].

### 2.3. Derivation of 95% CL Using Three Methods

Initially, the entire dataset was used to characterize the distribution of the PB_max_ values as a function of PTA, and to assess how accurately the 95% CLs were estimated for each PTA group using the simulation and HD methods.

### 2.4. Distribution of PB_max_ Values as a Function of PTA

**Figure 1:**
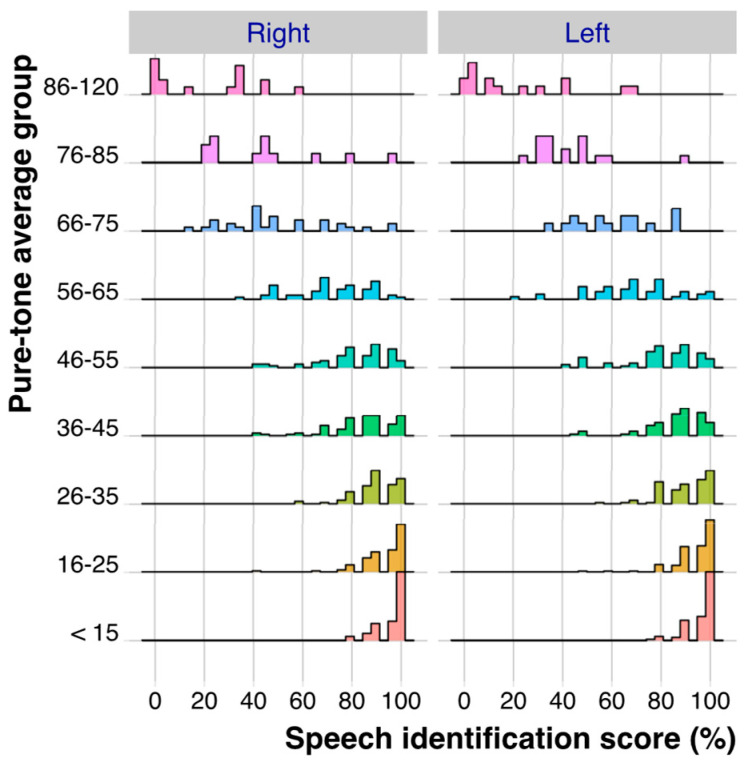
Distribution of the PB_max_ values for each PTA group, separately for the right ear (**left side**) and left ear (**right side**).

The PB_max_ and PTA values for the right and left ears were analyzed separately. To characterize the distribution of PB_max_ as a function of PTA, the PTA values were assigned to sub-groups: <15, 16–25, 26–35… The distributions of the PB_max_ values for each PTA sub-group for the left and right ears are shown in Figure 1. The PB_max_ values were highly skewed for several of the PTA sub-groups for both ears. For PTAs up to 55 dB HL, many PB_max_ scores were higher than 90%. The distributions were generally similar for the left and right ears, except for the PTA group 66–75 dB HL. The left-most columns of Table 1 and Table 2 present the mean PTA and number of ears for each PTA group, and, for PB_max_, the mean, SD, skewness, and kurtosis for the right ear (Table 1) and the left ear (Table 2).

### 2.5. Method 1: Deriving the Means and 95% CL for PB_max_ Using the Simulation Method

The means and 95% CL_S_ values were derived using a modification of the simulation method of Dubno, Lee, Klein, Matthews, and Lam [7] separately for the left and right ears. The PB_max_ scores within each PTA group were assumed to be normally distributed for the purpose of the computer simulations, as was done by Dubno et al. [7], even though the distributions clearly deviated from normal. A normal distribution with parameters mean and SD corresponding to the measured values for right and left ears shown in Table 1 and Table 2, respectively, was used to generate simulated data for each PTA sub-group using 50,000 simulations per sub-group. Note that the SDs here were estimated directly from the data, whereas Dubno et al. estimated SDs as 1.62 times the SDs estimated from the binomial distribution. The simulated PB_max_ values were set to 100 if they were >100 and were set to 0 if they were less than 0. The PB_max_ value below which 2500 or 5% of 50,000 runs fell for each PTA group was taken as the 95% CL_S_. Since the PB_max_ scores were obtained using lists of 25 nonsense syllables, when expressed as percentages, the scores were integer multiples of 4. Since the 95% CL_S_ values estimated using the simulation method were not the integer multiples of 4, the values obtained were rounded to the closest integer multiple of 4.

The means, SDs, and 95% CL_S_ values are shown in the middle columns of Table 1 and Table 2, for right and left ears, respectively. The simulated mean values differed slightly from the measured mean values, mainly because the simulated data were truncated when they were outside the range of 0–100%. The discrepancies in the SDs were larger, but not dramatically so.

### 2.6. Method 2: Deriving the Median and 95% CL for PB_max_ Using the HD Method [16]

The median (50% CL_HD_) and 95% CL_HD_ were estimated using the HD method [16] from bootstrapped samples using the procedure detailed by Harris and Boyd [15] and Wilcox [14]. The resulting values for each PTA group are shown in the right-most columns in Table 1 and Table 2.

In addition to 50% CL_HD_ and 95% CL_HD_, the standard errors (SEs) of the 95% CL_HD_ values were estimated using the bootstrap approach described by Harris and Boyd [15]. The SEs of the 95% CL_S_ values were estimated using the procedure described by Dowd [33]. The outcomes are shown in Table 1 and Table 2. The SEs of the 95% CL_S_ values were consistently higher than the SEs of the 95% CL_HD_ values.

### 2.7. Fitting the Means or Medians and 95% CL Values as a Function of PTA for the Simulation and HD Methods

To estimate the means, medians, and 95% CLs derived using the simulation and HD methods as continuous functions of PTA, it was necessary to select a function with a suitable general form. The function used by Dubno, Lee, Klein, Matthews, and Lam [7] fitted their data well for the range of PTA values that they considered (up to about 70 dB HL), but flattened off for larger PTA values, whereas our data indicated that the PB_max_ values approached zero quite steeply as the PTA approached 90 dB HL. Several functions were tried, and it was found that good fits could be obtained with the following function:

(1) $${PB}_{max}=\beta_{1}\times \left(2-e^{\left(\beta_{2\;}\times {PTA}^{\beta_{3}}\right)}\right)$$

where *β*_1_ is the value of PB_max_ for PTA = 0 dB HL, and *β*_2_ and *β*_3_ are the constants that determine the slope and curvature of the function with increasing PTA. For the values of PTA less than zero, PB_max_ was set to *β*_1_. When Equation (1) called for a value of PB_max_ less than zero, PB_max_ was set to zero.

### 2.8. Method 3: Non-Linear Quantile Regression

For nQR, the function relating PB_max_ to PTA as a continuous independent variable was similar in form to Equation (1), as specified by Equation (2).

(2) $$Y_{i}=\beta_{1}\times \left(2-e^{\left(\beta_{2\;}\left[\tau \right]\times {x_{i}}^{\beta_{3}\left[\tau \right]}\right)}\right)+e_{i}$$

where Y*_i_* is the *i*th observation of PB_max_, *xi* is the *i*th observation of the PTA, τ denotes the percentile, *β*_1_ is the value of PB_max_ for PTA = 0 dB HL, and *β*_2_ [τ] and *β*_3_ [τ] are the constants that determine the slope and curvature of the function with increasing PTA. The error terms, *e_i_*, were assumed to have a normal distribution with zero mean: $e_{i\;}\sim \;N(0,\sigma_{e}^{2})$. An interior point algorithm [19] was used for fitting the quantiles τ = 0.5 (Median), and τ = 0.05 (i.e., lower boundary, corresponding to the one-tailed 95% CL). For values of PTA less than zero, PB_max_ was set to *β*_1_. When Equation (2) called for a value of PB_max_ less than zero, PB_max_ was set to zero.

The goodness of fit of a nQR model is measured by *R*^1^(*τ*) [34,35], which is an analog of *R*^2^ for linear regression. *R*^1^(*τ*) is defined by Equation (3).

(3) $$R^{1}\left(\tau \right)=\frac{\sum_{i=1}^{n}\tau \left(y_{i}-{ý}_{i\;}\left(\tau \right)\right)}{\sum_{i=1}^{n}\tau \left(y_{i}-{y\left(\tau \right)}_{\;}\right)}$$

where *y*(*τ*) is the *τ*th quantile of the sample distribution of *y*, ${ý}_{i}\left(\tau \right)$ is the predicted PB_max_ value from the nQR model, $y_{i}$ represents the measured value of PB_max_, and *n* is the number of samples.

### 2.9. Evaluation of the Three Methods Using Random Draws

The initial evaluation of the accuracy of each method was based on the entire dataset. The measure of accuracy was the percentage of cases falling below the estimated 95% CL, which should be close to 5% if a method is accurate. To provide an additional measure of accuracy and to assess consistency, a random sample of 50% of the data (i.e., 321 participants), denoted the fitting set, was taken and was fitted using each method. Then, the fitted parameters were applied to the remaining 50% of the data, the test set, to estimate the 95% CL values for an independent dataset. This process was repeated 25 times using different random draws of 50% of the data. The deviation (DEV) of the percentage of cases falling below the fitted 95% CL from the target value of 5% was determined. The mean of DEV across the 25 draws gives a measure of the accuracy and the SD of the DEV values gives a measure of the consistency.

## 3. Results

**Figure 2:**
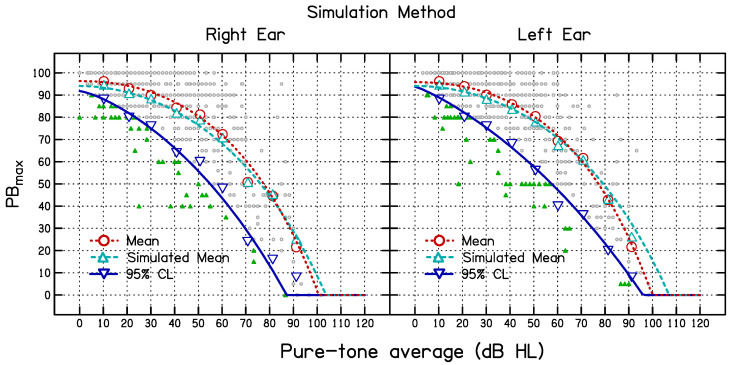
Results using the simulation method for the right ear (**left side**) and the left ear (**right side**). The large symbols show the measured means (circles) and simulated means (up-pointing diamonds) and simulated lower 95% CL_S_ (down-pointing triangles) for each PTA sub-group. The long-dashed curves and short-dashed curves show functions fitted to the measured and simulated means, respectively, for the nine PTA sub-groups. The solid curves show functions fitted to the 95% CL_S_ values for the nine PTA sub-groups. The small green filled triangles show individual PB_max_ values below the 95% CL_S_ and small open circles show PB_max_ values above the 95% CL_S_.

**Figure 3:**
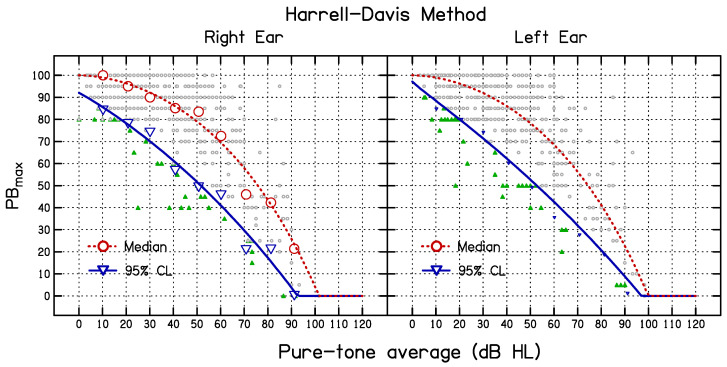
Median and 95% CL_HD_ values estimated using the Harrell–Davis method for the right ear (**left side**) and the left (**right side**). The large filled symbols show the medians (circles) and lower 95% CL_HD_ values (down-pointing triangles) for each PTA sub-group. The dashed curves show the functions fitted to the medians for the nine PTA sub-groups. The solid curves show the functions fitted to the 95% CL_HD_ values for the nine PTA sub-groups. The small green filled triangles show individual PB_max_ values below the estimated 95% CL_HD_ curve and small open circles show PB_max_ values above the 95% CL_HD_ curve.

The mean values of PB_max_ for the measured and simulated data (based on the entire dataset), and the 95% CL_S_ for the simulated data, were estimated as continuous functions of PTA using Equation (1) for both the simulation and HD methods by finding the values of *β*_1_, *β*_2_, and *β*_3_ that minimized the mean square deviation between the fitted functions and the simulated means. The resulting 95% CL values are shown in Table 1 and Table 2. The best-fitting parameter values are given in Table 3. The values of *β*_1_ and *β*_3_ were similar for the two ears but the values of *β*_2_ differed somewhat across the ears. The goodness of fit, measured by the proportion of variance accounted for, *R*^2^, is also shown in Table 3. The curves in Figure 2 show the best-fitting functions (long dashed—mean measured data; short dashed—mean simulated data; solid—estimated 95% CL_S_). The curves in Figure 3 show the best-fitting functions (dashed—median; solid—estimated 95% CL_HD_).

**Figure 4:**
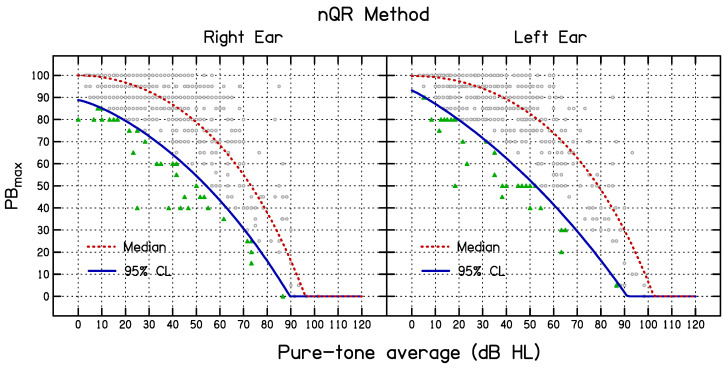
Results using the nQR method for the right ear (**left side**) and the left ear (**right side**). The dashed lines show the fitted median and the solid lines show the fitted lower 95% CL_QR_. The small green filled triangles show individual PB_max_ values below the estimated 95% CL and the small open circles show PB_max_ values above the 95% CL.

The fitted nQR functions for the median and the 95% CL_QR_ values are shown in Figure 4. The estimated parameters, standard error of the parameters, and R^1^ values of the nQR model are given in Table 3. Note that the R^1^ values are not comparable to the R^2^ values for the simulation and HD methods, because the former are based on the fits to the data for the individual participants whereas the latter are based on the fits to the measured and simulated values for the PTA sub-groups.

### 3.1. Comparison of the 95% CL Predicted Using the Three Methods for All Data

**Figure 5:**
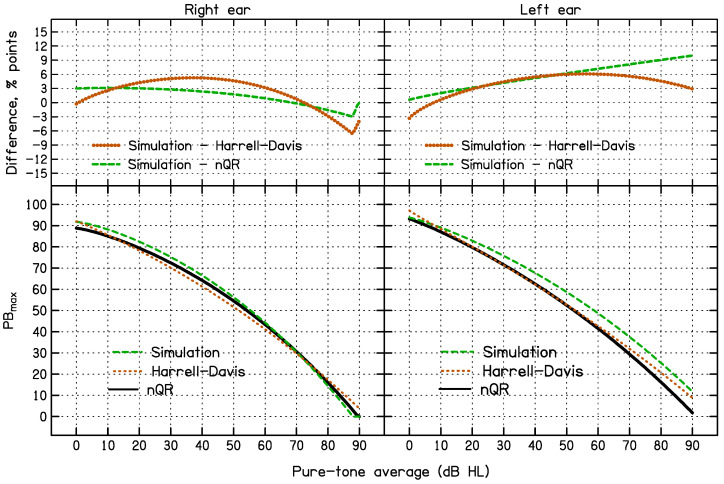
The lower panels compare the lower 95% CL estimated using the simulation method (dashed green line), the Harrell–Davis method (red dotted line), and the nQR method (black solid line) for the right ear (**left side**) and the left ear (**right side**). The upper panels show the differences between the simulation method and the two other methods.

The lower left and right panels of Figure 5 compare the 95% CL as a function of PTA estimated using the simulation, HD, and nQR methods including all the data. The 95% CLs were very similar for the HD and nQR methods. The upper panels show the differences between the simulation and HD methods and the simulation and nQR methods. Compared to the HD and nQR methods, the simulation method led to slightly higher 95% CL values for low to medium PTA values for the right ear and for medium to high PTA values for the left ear. As a result, a greater percentage of the ears fell below the estimated 95% CLs for the simulation method than for the HD or nQR methods. For the simulation method, 6.8% and 6.7% of the PB_max_ values fell below the 95% CL_S_ curve for the right and left ears, respectively, values that are above the target value of 5%. In contrast, for the HD method, 4.9% of the PB_max_ values fell below the 95% CL_HD_ curve for both right and left ears and for the nQR method, 5.3% of the PB_max_ values fell below the 95% CL_QR_ curve for both right and left ears, both close to the desired value of 5%.

### 3.2. Accuracy and Consistency of the 95% CL Predicted Using the Three Methods

**Figure 6:**
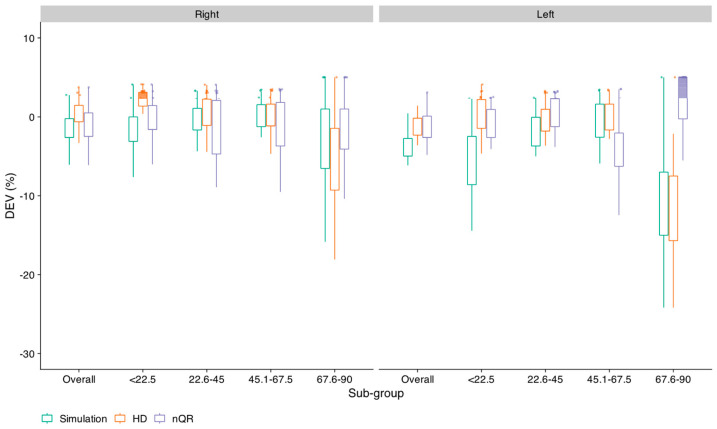
Box plots of the difference (DEV) between the percentage of cases falling below the estimated 95% CL for the test dataset and the target percentage of 5% for each of the three methods. The results are shown for the overall data and for each PTA sub-group, separately for the right ear (**left side**) and left ear (**right side**).

This section presents the results of the analyses based on fitting the data for a random sample of 50% of the total data (the fitting set) and then assessing the accuracy and consistency of the predictions for the test set, the whole procedure being repeated 25 times using different randomly selected draws. The key measure was the difference between the percentage of cases falling below the estimated 95% CL for the test dataset and the target percentage of 5%, denoted DEV. The smaller the mean value of DEV, the more accurate the method. The smaller the SD of DEV, the more consistent the method. Figure 6 shows box plots of the values of DEV for each method and each ear, separately for each PTA sub-group and overall. Each small circle shows the results for one random draw of the fitting and test sets. DEV values falling below 0 indicate that the proportion of the PB_max_ values falling below the estimated 95% CL is greater than the target value of 5%.

Overall, the simulation method showed the poorest accuracy. The HD and nQR methods showed similar accuracy, except for the PTA sub-group with the highest PTA, for which the nQR method showed better accuracy than the HD method, especially for the left ear. A mixed analysis of variance (ANOVA) was performed on the DEV values with ear and method as within-subject factors and the PTA sub-group as a between-subject factor. The Greenhouse–Geissler correction was applied to the degrees of freedom when the condition of sphericity was not met, but the uncorrected degrees of freedom are reported. There were significant main effects of method [F_(2, 192)_ = 18.1, *p* < 0.0001, $\eta^{2}$ = 0.045], ear [F_(1, 96)_ = 34.0, *p* < 0.0001, $\eta^{2}$ = 0.036] and PTA sub-group [F_(3, 96)_ = 16.7, *p* < 0.0001, $\eta^{2}$ = 0.32]. There were significant two-way interactions of ear and method [F_(2, 192)_ = 13.8, *p* < 0.001, $\eta^{2}$ = 0.033], method and PTA sub-group [F_(6, 192)_ = 29.7, *p* < 0.0001, $\eta^{2}$ = 0.189], and ear and PTA sub-group [F_(3, 96)_ = 7.1, *p* < 0.0001, $\eta^{2}$ = 0.023]. The three-way interaction of method, PTA sub-group, and ear was also significant [F_(6, 192)_ = 11.1, *p* < 0.0001, $\eta^{2}$ = 0.077]. The main interest here is the effect of the method. Averaged across PTA sub-groups and ears, the values of DEV were −3.2, −1.8, and −1.1% for the simulation, HD, and nQR methods, respectively. Pair-wise comparisons with Bonferroni correction showed that there were significant differences between the simulation method and the HD (t = −3.7, *p* < 0.001) and nQR (t = −5.9, *p* < 0.001) methods, but no significant difference between the nQR and HD methods (t = −2.1, *p* = 0.098).

A second ANOVA was conducted based on the overall DEV values with within-subject factors ear and method. This showed significant main effects of method [*F*_(2, 48)_ = 18.5, *p* < 0.0001, $\eta^{2}$ = 0.165] and ear [*F*_(1, 24)_ = 14.6, *p* < 0.001, $\eta^{2}$ = 0.093]. The two-way interaction between method and ear was also significant [*F*_(2, 48)_ = 4.9, *p* = 0.01, $\eta^{2}$ = 0.077]. Averaged across ears, the mean values of DEV were −2.7, −0.9, and −1.1 for the simulation, HD, and nQr methods, respectively. Pair-wise comparisons with Bonferroni correction showed that there were significant differences between the simulation method and the HD (*t* = −5.8, *p* < 0.001) and nQR methods (*t* = −4.3, *p* < 0.001), but no significant difference between the nQR and HD methods (*t* = 1.54, *p* = 0.12).

The SDs of the values of DEV provide a measure of consistency. Consider first the results for the PTA sub-groups. For the simulation method, the SD ranged from 2.3 to 6.3, with a mean of 3.9. For the HD method, the SD ranged from 1.9 to 6.6, with a mean of 3.1. For the nQR method, the SD ranged from 2.5 to 6.5, with a mean of 4.0. Based on variance-ratio (*F*) tests, the differences in SD across all the pairs of methods were not significant. For the overall data averaged across ears, the values of the SDs of the DEV values were 2.4, 1.6, and 2.2 for the simulation, HD, and nQr methods, respectively. The differences in SD across all the pairs of methods were not significant.

It can be concluded that the three methods had similar consistency but both the HD and nQR methods had greater accuracy than the simulation method. The HD and nQR methods showed similar accuracy, but the nQR method gave smaller DEV values for the PTA sub-group 67.6–90 dB HL, which had the smallest number of participants. Thus, the nQr method is preferable over the HD method.

### 3.3. Effect of Sub-Group Formation on the Outcome of the Simulation and HD Methods

The results of the simulation and HD methods depend on how the ears are assigned to PTA groups, whereas the nQR method does not depend on the formation of sub-groups. To assess the effect of grouping criteria on the 95% CL obtained using the simulation and HD methods, four sets of PTA groups were used, as shown in Table 4; these are denoted Group 1 to Group 4. The 95% CL_S_ and 95% CL_HD_ for each sub-group for each grouping method were generated and 95% CL was estimated as a continuous function of the PTA for each grouping method using Equation (1). These analyses were based on the entire dataset.

**Figure 7:**
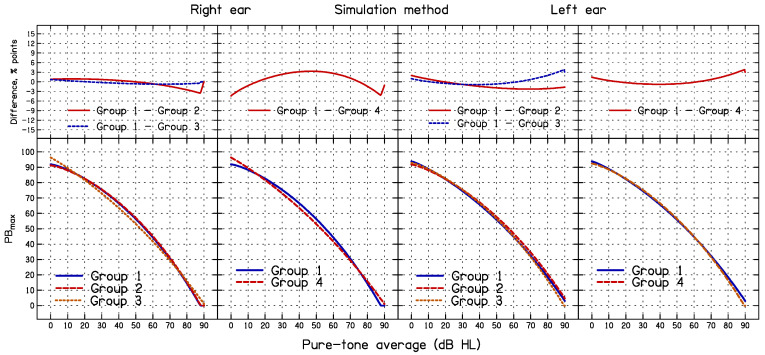
The lower panels show lower 95% CL_S_ estimated using the simulation method for the four PTA groups for the right ear (**left two panels**) and the left ear (**right two panels**). The upper panels show differences for specific pairs of PTA groups.

Fitted functions obtained using the simulation method for each of the four grouping methods are plotted in the lower panels of Figure 7, separately for the right and left ears. Group 1 is the same as used previously in the analysis of the simulation and HD methods. The differences between Group 1 and Groups 2–4 are plotted in the upper panels of Figure 7. The differences range from about −4 to 4 percentage points, with RMS differences ranging from 0.53 to 2.3 percentage points for the right ear, and from 1.2 to 1.6 percentage points for the left ear. This is unfortunate, because it is not obvious what grouping is the most appropriate one to use. For Groups 1 to 4, approximately 6.8%, 6.3%, 7.3%, and 7.8% of the PB_max_ values fell below the estimated 95% CL_S_, respectively, for the right ears and approximately 6.7%, 7.1%, 8.1%, and 8.2% of the PB_max_ values fell below the estimated 95% CL_S_ for the left ears. Thus, regardless of grouping, the percentage of PB_max_ values below the estimated CL_S_ values was greater than the target value of 5%.

**Figure 8:**
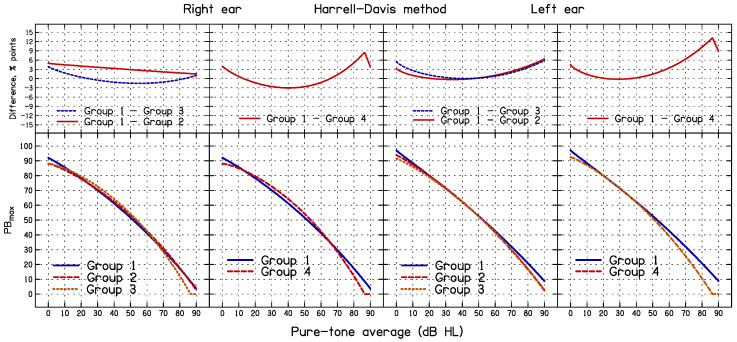
The lower panels show lower 95% CL_HD_ estimated using the Harrell–Davis method for the four PTA groups for the right ear (**left two panels**) and the left ear (**right two panels**). The upper panels show differences for specific pairs of PTA groups.

The fitted functions obtained using the HD method for each of the four PTA grouping methods are plotted in the lower panels of Figure 8, separately for the right and left ears. The differences between Group 1 and Groups 2–4 are plotted in the upper panels of Figure 8. The differences range from about −3 to 10 percentage points, with the RMS differences ranging from 2.0 to 3.7 percentage points for the right ear, and from 1.3 to 3.0 percentage points for the left ear. For Groups 1 to 4, approximately 5.4%, 5.3%, 4.9%, and 5.3% of the PB_max_ values fell below the estimated 95% CL_HD_, respectively, for the right ears and approximately 4.9%, 4.8%, 4.9%, and 4.8% of the PB_max_ values fell below the estimated 95% CL_HD_ for the left ears. Thus, regardless of grouping, the percentage of PB_max_ values below the estimated CL_HD_ values was close to the target value of 5%.

## 4. Discussion

The first objective of this study was to compare the accuracy of the three methods. The 95% CL values estimated using HD and nQR methods were reasonably accurate overall, since for the entire dataset, approximately 5% of the PB_max_ values fell below the estimated 95% CL values for PTA values up to 76 dB HL, and for the analysis based on 25 random draws of 50% of the data, the DEV values were close to zero; mean values of DEV were −1.8 and −1.1% for the HD and nQR methods, respectively. For the simulation method, the percentage of the PB_max_ values falling below the estimated 95% CL values was greater than the target value of 5%; the accuracy was poorer. The mean value of DEV was −3.2 (corresponding to 8.2% of the cases falling below the estimated 95% CL_S_), more than double the value for the HD and nQR methods. The relatively poor accuracy of the simulation method may be related to the sub-grouping, to the small number of samples in some sub-groups, and to the observation that the PB_max_ values within each PTA group were often not normally distributed [17,20,36].

It should be noted that Dubno, Lee, Klein, Matthews, and Lam [7] reported that about 5% of the PB_max_ values fell below the estimated 95% CL_S_ values. The difference from our results may be related to the sub-groups used; Dubno et al. used the PTA groups of variable width so as to obtain a reasonable number of participants within each group, but even so, some groups had a relatively small number of participants. Small numbers in each group can result in sampling errors and inaccurate estimates of a variety of measures [37,38,39]. Another possible contributor to the difference is that the SDs of the PB_max_ values here were estimated directly from the data, whereas Dubno et al. estimated SDs as 1.62 times the SDs estimated from the binomial distribution.

The second objective of the present study was to compare the consistency of the three methods. This was accomplished by comparing the SDs of the DEV values for the three methods. The SDs did not differ significantly across methods, indicating similar consistency. The third objective was to assess the effect of the formation of sub-groups, which is required for the simulation and HD methods. For the simulation method, the 95% CL_S_ values depended somewhat on the way that the sub-groups were formed. While the overall percentage of ears falling below the 95% CL_S_ curve did not vary markedly depending on the grouping method, the shape of the 95% CL_S_ curve did vary with the grouping method, the difference being most pronounced between Groups 1 and 4 (see Figure 7). For the HD method, the shapes of the 95% CL_HD_ curves also depended somewhat on the sub-group formation (see Figure 8). This is problematic, as there is no obvious way of deciding on the optimal grouping. The nQR method has the advantage that it does not depend on the arbitrary formation of sub-groups.

Overall, the nQR method seems to be preferable to the simulation or HD methods because (1) the nQR method does not depend on the formation of PTA sub-groups; (2) the percentage of ears falling below the 95% CL_QR_ curve was close to 5% for the nQR method, whereas it was above 5% for the simulation method.

In diagnostic audiology, PB_max_ scores may be useful for identifying specific disorders, such as central disorders or auditory neuropathy. For example, Rance [40] compared the PB_max_ scores of participants with auditory neuropathy as a function of PTA with the norms provided by Yellin, Jerger, and Fifer [11]. Approximately 60% of the participants had PB_max_ scores below the norms. It may also be the case that changes in PB_max_ scores over time are more effective than HTLs in identifying changes in hearing resulting from an underlying disorder.

Unaided PB_max_ scores can provide useful information for the fitting of hearing aids and for counseling patients and their relatives about the likely benefits of hearing aids. Kirkwood [41] reported that 92% of the audiologists surveyed used some type of speech audiometric procedure. It is generally believed that individuals with hearing loss who have high PB_max_ scores are likely to derive good benefits from the amplification provided by hearing aids, whereas those with very low PB_max_ scores are likely to gain lower benefits, or at least to achieve lower overall aided performance. Some investigators have shown that hearing aid usage and satisfaction are significantly related to unaided PB_max_ values [42,43,44,45], whereas other investigators have shown no or only a weak relationship [46,47,48,49].

Most studies assessing the relationship of unaided PB_max_ scores with hearing aid outcome measures have employed simple correlation or regression to assess the relationship. It might be more appropriate to employ nQR for studying the relationship. For example, individuals could be classified based on the quantile range that their PB_max_ scores fall into given their PTA, for example, above 75%, between 75% to 50%, between 50% to 25%, and falling below 25%. It can be expected that those who fall in the top quartile will gain considerable benefit from hearing aids, while those falling in the bottom quartile will receive less benefit.

## 5. Conclusions

The nQR and HD methods are more accurate than the simulation method in estimating the lower 95% CL for PB_max_ as a function of PTA. Furthermore, the nQR method does not depend upon the division of participants into sub-groups based on their PTA. Based on the nQR method, for the Kannada nonsense syllables used here, the lower 95% CL for PB_max_ is about 86% correct when the PTA is 0 dB HL, 55% when the PTA is 50 dB HL, and 27% when the PTA is 70 dB HL.

## Figures and Tables

**Table 1:** The table shows, for each PTA group, the number of participants (*N*), and for the right ear only, the mean PTA, and, for the PB_max_ values, the mean, SD, skewness, and kurtosis for the measured data, and the mean, SD, 95% CL, standard error (SE) of the 95% CL, and the percentage of PB_max_ values below each 95% CL value for the simulation method and the Harrell–Davis method.

PTA Range	*N*	PTA (dB HL)	Measured Data				Simulation Method					Harrell–Davis			
			Mean	SD	Skewness	Kurtosis	Mean	SD	95% CL_S_	SE of 95% CL_S_	Percent below 95% CL_S_	Median	95% CL_HD_	SE of 95% CL_HD_	Percent below 95% CL_HD_
<15	151	10.2	96.2	5.6	−1.3	3.8	94.6	4.1	88	0.95	9.3	100.0	84.1	0.15	3.7
16–25	115	20.8	93.0	8.9	−2.4	13.1	90.8	6.6	80	1.8	3.5	95.0	78.0	0.45	3.6
26–35	96	30.1	89.9	8.4	−1.0	4.7	88.4	6.6	76	1.8	6.3	90.0	74.1	0.46	3.4
36–45	91	40. 8	84.3	13.2	−1.2	4.9	82.0	10.2	64	2.9	6.6	85.1	56.8	0.48	4.5
46–55	69	50.7	81.2	14.4	−1.2	4.1	78.9	11.9	60	3.7	7.3	83.5	49.3	0.19	5.5
56–65	58	60.2	72.3	15.3	−0.4	2.3	70.9	12.3	48	4.3	5.2	72.5	45.7	0.32	5.1
66–75	32	70.8	50.8	21.7	0.4	2.3	50.8	16.0	24	8.1	3.1	46.0	20.9	0.17	6.3
76–85	14	81.3	44.6	23.5	0.9	2.8	45.4	16.5	16	13.8	0.0	42.3	21.1	0.19	15.4
86–120	16	91.2	21.6	20.5	0.3	1.7	25.3	14.5	8	10.8	13.3	21.4	0.1	0.0	13.3

**Table 2:** The table shows, for each PTA group, the number of participants (*N*), and for the left ear only, the mean PTA, and, for PB_max_ values, the mean, SD, skewness and kurtosis for the measured data, and the mean, SD, 95% CL, standard error (SE) of the 95% CL, and the percentage of PB_max_ values below each 95% CL value for the simulation method and the Harrell–Davis method.

PTA Range	*N*	PTA (dB HL)	Measured Data				Simulation Method					Harrell–Davis			
			Mean	SD	Skewness	Kurtosis	Mean	SD	95% CL_S_	SE of 95% CL_S_	Percent below 95% CL_S_	Median	95% CL_HD_	SE of 95% C_HD_	Percent below 95% CL_HD_
<15	126	12.1	96.2	5.5	−1.5	5.0	94.6	4.1	88	1.1	6.3	100.0	84.7	0.06	4.0
16–25	147	19.9	93.9	7.8	−2.2	10.9	91.9	5.8	80	1.4	8.2	95.0	79. 9	0.45	3.4
26–35	104	30.3	90.1	9.3	−0.9	3.9	88.2	7.2	76	1.9	4.8	91.4	74.1	0.38	4.8
36–45	83	40.8	85.8	11.8	−1.5	5.6	83.7	9.2	68	2.7	6.0	89.0	60.1	0.25	4.8
46–55	84	50.6	80.4	14.7	−1.0	3.5	78.0	11.5	56	3.4	10.7	82.5	49.1	0.52	3.5
56–65	48	61.2	69.3	18.0	−0.5	3.3	67.4	14.0	40	5.5	6.3	70.0	35.3	0.55	6.3
66–75	16	70.6	61.6	16.2	0.0	1.9	60.8	13.5	36	8.6	6.3	61.4	27.6	0.09	6.3
76–85	18	80.7	42.8	15.6	1.6	5.7	43.2	13.3	20	7.7	0.0	37.7	18.6	0.03	5.6
86–120	15	91.3	21.7	22.9	1.0	2.8	26.2	15.7	8	12.5	13.3	21.2	1.1	0.00	13.3

**Table 3:** Parameter values (with standard errors in parentheses) of the functions fitted to the mean measured data, the mean simulated data, and the simulated 95% CL for each PTA group using the simulation method, and the median and 95% CL for the Harrell–Davis and the nonlinear QR methods. R^2^ and R^1^ values are measures of the goodness of fit. The results for the right and left ears are shown in the top and bottom halves, respectively.

	Right Ear													
	Simulation method				Harrell–Davis					Nonlinear QR				
	*β*_1_	*β*_2_	*β*_3_	R^2^		*β*_1_	*β*_2_	*β*_3_	R^2^		*β*_1_	*β*_2_	*β*_3_	R^1^
Mean (Measured)	96.3 (2.7)	0.0000312 (0.000051)	2.17 (0.27)	0.98										
Mean (Simulated)	94.1 (2.7)	0.0000978 (0.00018)	1.91 (0.27)	0.98	Median	100 (4.37)	0.000154 (0.00023)	1.82 (0.34)	0.97	Median	100 (1.2)	0.0000900 (0.000060)	1.96 (0.15)	0.67
95% CI_S_	91.8 (6.9)	0.00182 (0.0024)	1.33 (0.31)	0.97	95% CL_HD_	92.07 (7.73)	0.00613 (0.00063)	1.04 (0.23)	0.98	95% CL_QR_	88.8 (4.6)	0.00216 (0.0030)	1.29 (0.36)	0.76
	**Left Ear**													
	**Simulation method**				**Harrell–Davis**					**Nonlinear QR**				
	***β*_1_**	***β*_2_**	***β*_3_**	**R^2^**		***β*_1_**	***β*_2_**	***β*_3_**	**R^2^**		***β*_1_**	***β*_2_**	***β*_3_**	**R^1^**
Mean (Measured)	95.8 (1.9)	0.0000241 (0.000012)	2.23 (0.10)	0.99										
Mean (Simulated)	94.1 (1.3)	0.0000694 (0.000044)	1.97 (0.14)	0.98	Median	100 (4.53)	0.000159 (0.00024)	1.82 (0.34)	0.97	Median	99.7 (0.8)	0.0000500 (0.00001)	2.06 (0.05)	0.64
95% CI_S_	93.8 (3.9)	0.00348 (0.0021)	1.16 (0.14)	0.99	95% CL_HD_	97.1 (4.68)	0.0103 (0.0051)	0.92 (0.11)	0.99	95% CL_QR_	93.2 (5.1)	0.00536 (0.0057)	1.08 (0.24)	0.78

**Table 4:** PTA groups based on four sub-grouping criteria. The table shows: the number of participants (*N*) in each PTA group and the mean PTA for each group in dB HL for the right ears (top) and left ears (bottom).

**Right Ear**											
**Group 1**			**Group 2**			**Group 3**			**Group 4**		
**PTA Range**	***N***	**Mean**	**PTA Range**	***N***	**Mean**	**PTA Range**	***N***	**Mean**	**PTA Range**	***N***	**Mean**
<15	151	10.4	<10	78	7.5	<10	78	7.5	<15	151	10.4
16–25	115	20.8	11–20	136	15.8	11–15	73	13.5	16–30	169	23.1
26–35	96	30.4	21–30	106	26.0	16–20	63	18.4	31–45	133	38.4
36–45	91	40.7	31–40	89	36.0	21–25	52	23.7	46–60	104	53.0
46–55	69	50.5	41–50	79	45.3	26–30	54	28.2	61–75	55	67.6
56–65	58	60.2	51–60	69	55.6	31–35	42	33.4	76–120	30	86.6
66–75	32	70.7	61–70	41	65.7	36–40	47	38.4			
76–85	14	81.6	71–80	20	74.3	41–45	44	43.1			
86–120	16	90.8	81–120	24	88.6	46–50	35	48.0			
						51–55	34	53.0			
						56–60	35	58.0			
						61–70	41	65.7			
						71–80	20	74.3			
						81–120	24	88.6			
**Left Ear**											
**Group 1**			**Group 2**			**Group 3**			**Group 4**		
**PTA Range**	***N***	**Mean**	**PTA Range**	***N***	**Mean**	**PTA Range**	***N***	**Mean**	**PTA Range**	***N***	**Mean**
<15	126	12.1	<10	30	8.1	<10	30	8.1	<15	126	12.1
16–25	147	19.9	11–20	189	15. 6	11–15	96	13.3	16–30	209	22.4
26–35	104	30.3	21–30	116	25.9	16–20	93	17.9	31–45	125	38.2
36–45	83	40.6	31–40	95	36.4	21–25	54	23.4	46–60	107	52.4
46–55	84	50.6	41–50	76	46.5	26–30	62	28.2	61–75	41	66.2
56–65	48	61.2	51–60	61	55.4	31–35	42	33.5	76–120	34	85.0
66–75	16	70.6	61–70	33	64.4	36–40	53	38.8			
76–85	19	80.7	71–80	19	76.1	41–45	30	43.7			
86–120	15	91.2	81–120	23	88.7	46–50	46	48.3			
						51–55	38	53.4			
						56–60	23	58.7			
						61–70	33	64.4			
						71–80	19	76.1			
						81–120	23	88.7			

## Data Availability

Data are available with the corresponding author mentioned in this research paper.
